# From diagnosis to daily life: A comparative pilot study on healthcare access and challenges for neurofibromatosis type 1 in public systems of Brazil and Portugal

**DOI:** 10.1016/j.clinsp.2026.100875

**Published:** 2026-02-12

**Authors:** Déborah Domeneghetti de Francisco, Isabela Mayá Wayhs Silva, Jorge M. Saraiva, Carlos Eduardo Steiner, Vera Lúcia Gil-da-Silva-Lopes

**Affiliations:** a Department of Medical Genetics and Genomic Medicine, Faculty of Medical Sciences, State University of Campinas (Unicamp), Campinas, SP, Brazil; b Medical Genetics Department, Hospital Pediátrico de Coimbra, Unidade Local de Saúde de Coimbra, Coimbra, Portugal; c University Clinic of Pediatrics, Faculty of Medicine, University of Coimbra, Portugal; d Clinical Academic Center of Coimbra, Hospital Pediátrico de Coimbra, Unidade Local de Saúde de Coimbra, Coimbra, Portugal

**Keywords:** Neurofibromatosis-1, Health services accessibility, Healthcare disparities, Health literacy, Cross-cultural comparison, Patient reported outcome measures

## Abstract

**Background:**

Neurofibromatosis type 1 is a systemic genetic disease with manifestations that include neurocognitive alterations. The disease's characteristics influence various areas of the lives of affected individuals, regardless of their place of residence.

**Objectives:**

This study compares the experiences of a small group of patients with type 1 neurofibromatosis in two public hospitals in Brazil and Portugal.

**Methods:**

This is an exploratory, cross-sectional, and descriptive study. Patients of legal age or those responsible for underage patients were included in a health service in the Clinical Hospital of the State University of Campinas in Brazil and the Clinical Academic Center of Coimbra in Portugal. They answered an online form about quality of life and access to health.

**Results:**

18 participants were included, 13 Brazilians and 5 Portuguese. Most were female and had a mean age at diagnosis of 8.2-years. The mean age of Brazilians was 9.1-years for the first consultation with a geneticist and 15.5-years among Portuguese. The average number of professionals accessed was 4.5 among Brazilians and 3.8 among Portuguese.

**Limitations:**

Small sample size, restricted temporal aspect, convenience bias of participants with internet access, and self-declaration of data by participants.

**Conclusions:**

Both Brazilians and Portuguese participants with neurofibromatosis type 1 presented similar difficulties regarding access to health. The differences found between the participants from both countries focus on access to medical geneticists and their health literacy. Expanding studies would help identify care priorities related to the specificities of NF1 that could improve the quality of life of affected individuals.

## Highlights

• First study of NF1 patient experiences in the health systems of Brazil and Portugal.
• Portuguese patients showed lower genetic and health literacy than Brazilians.
• Access to neurologists and psychiatrists was a key barrier in both countries.
• Despite economic differences, both nations face similar barriers in multidisciplinary care.
• Findings highlight urgent need for national NF1 data and structured care pathways.

## Introduction

Neurofibromatosis Type 1 (NF1) is an autosomal dominant inherited disease characterized by the presence of pigmentary changes and neurofibromas, besides occasional features in other systems,^1^ presents with significant clinical heterogeneity, and affects about 1 in every 2500 individuals worldwide, regardless of ancestry or sex.^2^ Its management requires the recognition of its clinical characteristics through a physical examination, specialized evaluations, and complementary tests.^1^

The appropriate management of individuals with NF1 involves multidisciplinary care, including follow-up with general practitioners, pediatricians, dermatologists, neurologists, psychiatrists, psychologists, and others, permeating various areas of health care.^3^

In Brazil, patients and families can access health services through private or public services. The public service in Brazil is structured in a Unified Health System (“Sistema Único de Saúde”, SUS), established by the Organic Law of SUS 8080/90, which has among its principles the universality and comprehensiveness of care, being the exclusive source of health care for about 71.5 % of over 200 million Brazilians.^4^^,^^5^ Regarding rare diseases, the National Policy for Comprehensive Care for People with Rare Diseases was instituted in 2014, which defined the guidelines for the care of individuals with rare diseases in the SUS in the various instances of the Health Care Network, and is still under development.^6^ In Portugal, a European country with around 10 million inhabitants, the National Health Service (SNS) was created in 1979 by the Health Framework Law, through which the State guarantees the right to health protection.^7^ The NHS is tax-funded and covers all residents.^8^ According to the World Index of Healthcare Innovation, in 2024, around 70 % of the Portuguese population relied on the NHS.^9^ Specifically in relation to rare diseases, Portugal has adopted a national strategy integrated into the NHS with the aim of enabling comprehensive access to healthcare and with the designation of centers of expertise.^10^ Notably, recent European initiatives such as the “Action for Rare Diseases 2025‒2030: From Strategy to Person” document have been developed. This document outlines the key dimensions and challenges related to prevention and health promotion for individuals with rare diseases and their families, keeping the healthcare community and the public updated on the latest developments in Portugal's healthcare system.^11^

Despite their differences in population size, territorial area, and regional economic disparities (mainly regarding Brazil), both countries have predominantly public health systems under government administration, sharing the principle that the State must guarantee access to health for the population.^12^ However, neither country provides statistics on the prevalence of NF1 in its population, hindering the development of public policies and assessing actual health needs. The National Network of Rare Diseases, a Brazilian multicenter research project that seeks to understand the scenario of rare diseases in the country, reports a prevalence of approximately 2 % of NF1 among its participants.^13^^,^^14^

This exploratory pilot study aims to describe the experience of accessing health services in two public hospitals in Portugal and Brazil, focusing on a small group of individuals.

## Methods

### Study design

This study is a cross-sectional and descriptive analysis conducted in 2023. Participants diagnosed with NF1 aged 18-years or older at the time of this study, or parents/guardians of people under 18-years of age or cognitively impaired diagnosed with this disease, who attended the Clinical Hospital of the State University of Campinas (HC/Unicamp) in Brazil and the Clinical Academic Center of Coimbra in Portugal, were included. The population served at HC/Unicamp consists of residents of Campinas and surrounding municipalities referred from primary health care units. The same applies to the Hospital Center in Coimbra and nearby cities. All participants signed an informed consent form. The study was approved by the Ethics Committee of the Universidade Estadual de Campinas (Unicamp) (CAAE: 2477419.1.0000.5404) and the Centro Hospitalar e Universitário de Coimbra (OBS.SF.147-2022) and was conducted according to the guidelines of the Declaration of Helsinki. The sample was selected for convenience as it is a rare disease, and data collection was through the internet. All participants in the Portuguese sample had completed molecular diagnosis and were provided with written discharge reports. In Brazil, the diagnosis of NF1 is usually based on clinical features, and a genetic report was provided during the genetic counseling session to share information with other healthcare professionals about NF1. All individuals included in this study were subsequently investigated through whole genome sequencing in another research protocol and had their diagnosis molecularly confirmed (data not shown).

### Data collection

A form containing 59 questions was divided into a) Socioeconomic identification, b) Access to medical genetics services, c) Access to health network services, d) Literacy, and e) Social insertion. The participants recorded the answers anonymously from pre-defined multiple-choice alternatives or numerical values in an electronic form (Google Forms).

### Data analysis

The answers were organized to describe the characteristics of the sample. Frequency tables were made for categorical variables with values of absolute frequency (n) and percentage frequency (%), and for quantitative variables, descriptive measures including mean, median, and Standard Deviation (SD) were obtained. Fisher's exact test was used to compare proportions. Spearman's correlation coefficient evaluated the correlation between quantitative variables. The level of significance adopted for the statistical tests was 5 %. The graphs for interpreting the analyses obtained were prepared using the Microsoft Office 365 software.

## Results

This exploratory study involves two reference regions in genetics in their respective countries. Although more individuals are diagnosed with NF1 in both hospitals, participation is voluntary, so the researchers do not have control over the number of participants. The sample description is detailed in Table 1 (*n* = 18). In this sample, no participant received social assistance grants.

**Table 1:** Description of participants' socioeconomic data (*n* = 18).

Characteristic	Total	Brazil	Portugal	p-value
**N**	18	13 (72.2 %)	5 (27.8 %)	a
**Sex**				
F (%)	12 (66.7 %)	8 (61.5 %)	4 (80 %)	0.61481
M (%)	6 (33.3 %)	5 (38.5 %)	1 (20 %)	
**Age group**				
Teenager or adult	11 (61.1 %)	8 (61.5 %)	3 (60 %)	11
Parents or guardians	7 (38.9 %)	5 (38.5 %)	2 (40 %)	
**Employability**				
Teenager or adult	10 (90.9 %; *n* = 11)	7 (87.5 %; *n* = 8)	3 (66.7 %; *n* = 3)	11
Parents or guardians	4 (57.1 %; *n* = 7)	2 (40 %; *n* = 5)	2 (100 %; *n* = 2)	
**Income**				
Up to 2-minimum wages	10 (55.5 %)	5 (38.5 %)	5 (100 %)	
Up to 3-minimum wages	2 (11.1 %)	2 (15.5 %)	0	a
Higher 4-minimum wages	3 (16.7 %)	3 (23 %)	0	
He/she didn't want to answer	3 (16.7 %)	3 (23 %)	0	
**Means of transport to the place of medical follow-up**				
Own car	8 (44.4 %)	5 (38.5 %)	3 (60 %)	a
Public transport	7 (38.9 %)	6 (46.1 %)	1 (20 %)	
Private bus company	1 (5.5 %)	1 (7.7 %)	0	

a It was not possible to calculate the p-value by sample size. ^1^ Calculation by Fisher's exact test.

### Access to diagnostics

The age of suspected diagnosis among Brazilians was 7.6 (median: 3.25, SD: 9.7) years and 8.8 (median: 5, SD: 13.8) years. Among Portuguese, with an overall average of 7.9 (median: 5, SD: 10.6) years. The mean age at confirmation of diagnosis among Brazilians is 8.2 (median: 2, SD: 10.5) years of age and 8.8 (median: 5, SD: 13.8) years among Portuguese, with an overall mean age of 8.3 (median: 3.5, SD: 11.1) years.

For diagnostic confirmation, 8 (61.5 %) Brazilians reported a clinical diagnosis of the condition, while 5 (38.5 %) could not inform which test was used. Among the Portuguese, 4 (80 %) reported not knowing which test was used, and one Portuguese participant answered “None” to this question.

Regarding the evaluation with a geneticist, 12 (92.3 %) Brazilian participants reported having already had an appointment with this specialist, with a mean age of 9.1 years for the first consultation (median: 3.5, SD: 10.5). Among the Portuguese participants, 2 (40 %) had already had a consultation with a geneticist, with a mean age of 15.5 (median: 15.5, SD: 14.8) years. The mean age of the group was generally 10-years at the first visit (median: 4.5, SD: 10.8).

### Genetic literacy

All Brazilian participants answered that they knew what neurofibromatosis is, while 2 (40 %) Portuguese participants answered that they knew, and 3 (60 %) indicated that they knew partially (*p* = 0.0123 – Fisher's exact test). In the general sample, 14 (77.8 %) of the participants reported knowing or partially knowing the cause of their syndrome, of which 12/13 (92.3 %) were Brazilian, and 2/5 (40 %) were Portuguese. When asked if they knew which health problems are associated with NF1, 12 (92.3 %) of the Brazilian participants answered “yes” or “partially”, and 1 (7.7 %) answered “no”. In the Portuguese group, 3 (60 %) respondents were unsure about potential health issues associated with NF1, while 2 (40 %) reported having some knowledge.

The receipt of a positive report for the diagnosis was reported by 13 (72.2 %) participants, of whom 11/13 (84.6 %) were Brazilian, and 2/5 (40 %) were Portuguese (*p* = 0.0987; Fisher's exact test). On the other hand, the receipt of a written report explaining the health problems related to neurofibromatosis and how to treat them was reported by 8 (44.4 %) individuals, 7/13 (53.8 %) Brazilian and 1/5 (20 %) Portuguese (*p* = 0.3137; Fisher's exact test). This report was delivered to the follow-up physician by 11 (61.1 %) of the participants, 10/13 (76.9 %) Brazilian and 1/5 (20 %) Portuguese. Among other professionals, 10 (55.6 %) individuals reported having submitted the report, with 9/13 (69.2 %) from the Brazilian group and 1/5 (20 %) from the Portuguese group.

Both the participants who received and did not receive a written report explaining the problems associated with NF1 accessed a similar number of professionals, 4 (median: 3.5, SD: 2.3) among those who received a report and 4.8 (median: 4.5, SD: 2.5) among those who did not.

### Health monitoring

**Fig. 1:**
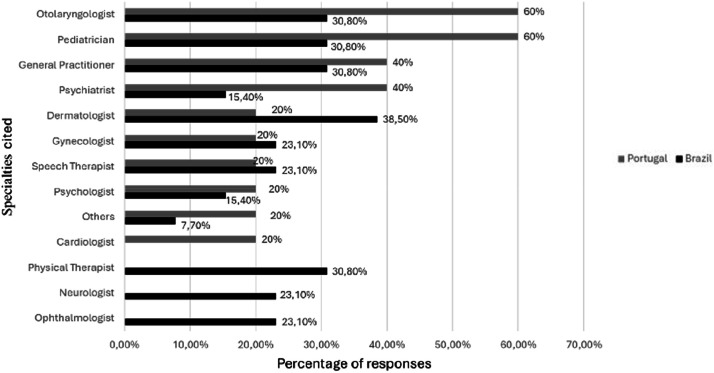
Specialties monitored.

Regarding the health follow-up of these individuals, 16 (88.9 %) of the participants reported being monitored in a health service, 12/13 (92.3 %) Brazilians and 4/5 (80 %) Portuguese. When asked about the place of care, 12 (92.3 %) Brazilian participants reported being seen at a Health Center by the SUS, including the participant who had answered that he was not being cared for, and one reported being seen in a private office. Among the Portuguese, 4 (80 %) reported receiving care at a public hospital, and one participant, who had answered that he was not being monitored, reported being treated at a Health Center. The average number of professionals accessed in the general group was 4.3 (median: 4, SD: 1.9), with an average of 4.5 (median: 4, SD: 2) among Brazilians and 3.8 (median: 5, SD: 1.8). The follow-up with specialists is shown in Fig. 1.

**Fig. 2:**
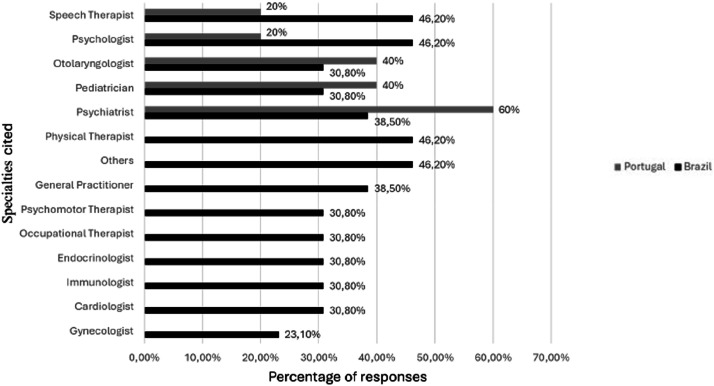
Specialties with bimonthly follow-up or higher.

**Fig. 3:**
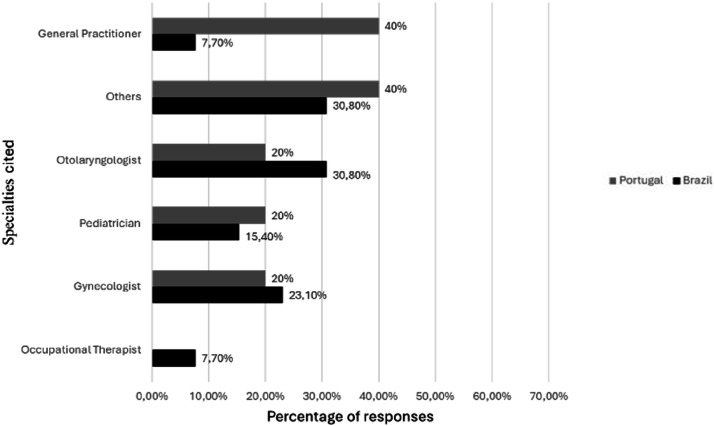
Specialties with follow-up less than bimonthly.

No specialty showed a difference in the number of participants undergoing follow-up between the two participant groups. Fig. 2, Fig. 3 show the specialties under follow-up in the two groups and their frequencies.

**Fig. 4:**
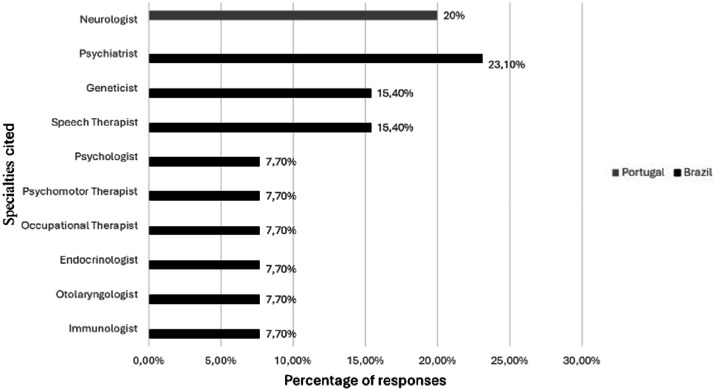
Specialties with difficult access.

There was no difference between the participants from the two countries regarding their frequency in the specialties. Difficulty in obtaining care in any specialty was reported by 7 (38.9 %) participants, of whom 6/13 (46.2 %) are Brazilian, and 1/5 (20 %) is Portuguese. Fig. 4 lists the specialties reported with this difficulty by the two groups.

The number of professionals accessed by unemployed individuals was, on average, 4.2 (median: 3, SD: 2.2). In contrast, employed individuals had access to 4.4 (median: 5, SD: 1.9) professionals on average. Regarding the difficulty of care, 30.8 % of the employed individuals reported difficulty accessing some specialties, while 60 % of unemployed individuals reported the same difficulty.

The age at confirmation of diagnosis did not influence the number of professionals accessed (*p* = 0.5979; Spearman's correlation coefficient). Participants who had difficulty receiving care in some specialty had a mean age at diagnostic confirmation of 5.1-years (median: 1, SD: 9.1), while those who did not have difficulty accessing had a mean age at confirmation of 10.4 (median: 5, SD: 12.2) years. Among the participants who undergo regular follow-up at a health service, the mean age at confirmation of diagnosis was 6.9 (median: 2, SD: 9.8) years, and among those who do not undergo follow-up, this average is 20 (median: 20, SD: 18.4).

Regarding the routine evaluation, 8 (44.4 %) participants reported having already undergone or had undergone cardiac assessment, 5/13 (38.5 %) Brazilian, and 3/5 (60 %) Portuguese (*p* = 0.6078; Fisher's exact test). Ophthalmologic evaluation is performed or has already been performed by 17 (94.4 %) individuals, 12/13 (92.3 %) Brazilians, and all 5 Portuguese participants. Hearing assessment was reported by 14 (77.8 %) participants, 11/13 (84.6 %) Brazilians, and 3/5 (60 %) Portuguese (*p* = 0.5327; Fisher's exact test).

Spinal evaluation (scoliosis) has already been performed or is performed by 6 (33.3 %) individuals, of which 5/13 (38.5 %) are from the Brazilian group, and 1/5 (20 %) is from the Portuguese group (*p* = 0.6148; Fisher's exact test). The evaluation with an orthopedist was reported by 7 (38.9 %) participants, 6/13 (46.2 %) Brazilian, and 1/5 (20 %) Portuguese (*p* = 0.5956; Fisher's exact test). Half of the participants reported having or having already undergone a kidney/abdominal ultrasound, a group composed only of participants from Brazil.

When asked about diagnoses of psychiatric disorders, 3 (16.7 %) reported having already received some diagnostic conclusion, 1/13 (7.7 %) Brazilian and 2/5 (40 %) Portuguese. However, when asked for details of the condition, 6 (33.3 %) reported some change, with 3/13 (23.1 %) Brazilians reporting anxiety and 3/5 (60 %) Portuguese reporting anxiety, depression, and hyperactivity, respectively; 3 (16.7 %) participants reported the use of psychiatric medications, 2/13 (15.4 %) Brazilian and 1/5 (20 %) Portuguese.

Regarding mental health care, 5 (27.8 %) participants reported receiving or having already received psychiatric care, of which 2/13 (15.4 %) are Brazilian, and 3/5 (60 %) are Portuguese (*p* = 0.0987; Fisher's exact test). Psychological care was reported by 7 (38.9 %) of the participants, 4/13 (30.8 %) Brazilians, and 3/5 (60 %) Portuguese (*p* = 0.3260; Fisher's exact test). Among the female patients, 8 (66.7 %) stated that they had been or had already been to a gynecologist, corresponding to 6/8 (75 %) in the Brazilian group and 2/4 (50 %) in the Portuguese group (*p* = 0.5475; Fisher's exact test). Regarding reproductive interest, 4 (22.2 %) of the participants reported an active sexual life, 3/13 (23.1 %) Brazilian and 1/5 (20 %) Portuguese. The use of contraceptive methods was reported by 2 (11.1 %) individuals, both Brazilians.

### Social and job integration

Regarding school insertion, half of the participants reported attending school, 6/13 (46.2 %) Brazilians and 3/5 (60 %) Portuguese. All parents and guardians reported that the children in their care attended school. The two adults/adolescents who reported attending school are a Brazilian participant with incomplete higher education and a Portuguese participant with complete higher education. Among the individuals who reported not attending school, 2/9 (22.2 %) had not completed high school, 3/9 (33.3 %) had completed high school, 1/9 (11.1 %) had completed higher education, and 3/9 (33.3 %) had a postgraduate degree.

All Portuguese participants attending school reported using regular schools. Among the Brazilians, 5/6 (83.3 %) participants who reported attending school reported using regular schools, and one reported attending a special needs school. Another 7 Brazilian participants reported attending regular school, although they answered negatively to “Do you attend school?”.

The need for a tutor or caregiver was reported by 2/6 (33.3 %) Brazilian participants, and no participant from the Portuguese group. Learning difficulties were reported by 7 (77.8 %) participants, of whom 5/6 (83.3 %) are from the Brazilian group and 1/3 (33.3 %) is from the Portuguese group. The need for a support room was reported by 7 (77.8 %) participants, corresponding to 5/6 (83.3 %) Brazilians and 1/3 (33.3 %) Portuguese, and the only one in this group informed that the school does not offer this support.

All these participants reported having informed the school about the diagnosis of their syndrome. Regarding acceptance by the other children, 4 (44.4 %) individuals reported having experienced some bullying situation, half of the Brazilian group and half of the Portuguese group.

Among the 11 adult and adolescent participants, 8 (72.3 %) reported job insertion, of which 6/8 (75 %) are Brazilian, and 2/3 (66.7 %) are Portuguese. The mean overall age at work initiation was 16.6-years (median: 16, SD: 0.9). The mean age among Brazilians was 16.5-years (median: 16, SD: 0.8); in the Portuguese group, it was 17-years (median: 17, SD: 1.4).

## Discussion

Neurofibromatosis type 1 is a multisystem disease that may include, among its most severe manifestations, involvement of the central nervous system, gastrointestinal and pulmonary tracts, cognitive deficits, scoliosis, bone dysplasias, and endocrinological disorders.^1^ Thus, it is necessary to longitudinally monitor the affected individuals in various instances of care for their full development.

In this context, given that Brazil and Portugal have distinct economic and territorial realities and similarly lack analyses regarding the frequency of NF1 in their populations, the exploratory observation of data from affected individuals may serve as a starting point for further research and for discussing their actual health needs and the healthcare attention required in each setting.

Although they are not directly related, social determinants, such as education, income, and housing, are essential for equitable access to health care.^12^ Portugal is a European country that makes up the economic bloc of the European Union and is included in the Eurozone.^15^ Despite the difficulties encountered during the COVID-19 pandemic, the Portuguese economy recovered strongly, with unemployment rates of around 6.6 %, compared with 14 % in the Brazilian reality.^12^^,^^16^ Even though it is the largest economy in Latin America, Brazil is considered a developing country, with socioeconomic indicators lower than those observed in Portugal, lacking greater public investments in infrastructure.^12^^,^^17^

Despite having similar unemployment and income rates in this study, with most participants receiving between 2 minimum wages, the economic realities of each country differentiate their purchasing power in each currency and, consequently, the quality of life of the families addressed. Even with this contextualization, employability did not influence the number of professionals accessed, which may indicate that the public health systems manage the care needs of these individuals well.

The mean age at diagnosis of the present sample was approximately 8-years, with no difference between the two groups and no influence on the number of professionals accessed. This age was found in a study conducted with centers in Saudi Arabia, which reported a mean of 8-years.^18^ A Brazilian study found a median age at diagnosis of 30-years in individuals with NF1 in its sample; however, it focused on an oncology sample, which may reflect a bias of this age-dependent feature.^19^ Another study that included 50 individuals from 30 unrelated families evaluated in the HC/Unicamp in the Brazilian center showed that the age at their first appointment ranged from 9-months to 62-years, with a mean of 21.7-years (unpublished data). Thus, there is no reliable data in the literature about diagnosis in NF1 in the Brazilian population. In the Portuguese context, a study reported a mean age at diagnosis of approximately 4-years in a neuropediatric center, which may be related to a more specialized evaluation.^20^

The observed age difference between Brazil and Portugal may be attributed to the inclusion criteria, since all Portuguese participants had their diagnoses confirmed through genetic testing requested by a medical geneticist. At the same time, another study considered clinical characteristics for diagnosis, which can go unnoticed. In both countries, there is a lack of national data on the prevalence of the disease, information that could help in understanding the population and its distribution throughout the territory to allocate more efficient resources.

The diagnosis of NF1 is made, in most cases, through clinical characteristics, such as café-au-lait spots, freckles in skinfolds, dermal neurofibromas, Lisch nodules, learning difficulties, behavioral changes, and skeletal abnormalities, as defined since 1988 and revised in 2021.^21^ The characteristics, however, are evolutionary and age-dependent, so most cases will only meet the criteria for this clinical diagnosis between 6- and 8-years of age, which is associated with a scenario of unpreparedness of other health professionals to identify signs that require a specialized genetic evaluation. This, combined with other health professionals' lack of training to identify signs that require specialized genetic evaluation, may be one of the factors related to late diagnosis and may compromise early treatment management and genetic counseling for parents, especially if additional genetic testing is not possible.^22^^,^^23^ There is a lower mean age of diagnosis in the sample among regularly followed-up participants, which may be justified by a better comparison of the professionals who participated in this care during the appearance of clinical signs with development, emphasizing the relevance of longitudinal follow-up.

When asked about consultation with a geneticist, the Portuguese group was less recognized concerning this evaluation, and at a higher age than the Brazilian group. Although geneticists treated all of them, this may be related to a deficiency of human resources involving the category in the country, which may generate a change in the follow-up of these patients after diagnosis, maintaining closer management of other professionals whom the Portuguese more cited.^24^ This difference may be due to participant perception, since the Portuguese may have established the timing of genetic counseling during adolescence rather than the confirmation test during the first appointment.

Furthermore, the Portuguese participants are less knowledgeable about their disease and have a lower rate of report delivery. Genetic literacy is the ability to understand, through concepts such as genes and DNA, the explanations regarding the genetic monitoring to which the individual is being submitted.^25^ The lack of this understanding can compromise the care process of these participants, from their investigation process to the possibility of making decisions consciously during genetic management and counseling.

In this context, preparing a report after diagnosis is also of great importance for communication between health professionals during multidisciplinary care, avoiding possible embarrassment for individuals with genetic diseases.^26^ Furthermore, the acknowledgment of the report contributed to participants' understanding, as all who received it reported an understanding of the health problems related to neurofibromatosis. Despite this, the supply of the report did not affect the number of professionals attending. All Portuguese participants had confirmed genetic diagnoses and were provided with written discharge reports. Despite this, the Portuguese participants did not acknowledge this care, which may be due to the absence of a medical geneticist during follow-up.

The most accessed professionals differed between the two groups, emphasizing otorhinolaryngologists in Portugal and dermatologists in the Brazilian sample. The least consulted professionals were cardiologists among Brazilians and neurologists and ophthalmologists among Portuguese. In the context of NF1, access to other health professionals linked to supportive therapies, such as speech therapists and psychologists, is recommended in the international management guide and is essential for establishing comprehensive and individualized care.^3^ Pediatricians, general practitioners, and psychiatrists were mentioned in both realities, emphasizing their importance in initial and continuous clinical management of the needs of this population, often as identifiers of the first manifestations of the disease.^3^

Despite its relevance, neurologists, psychiatrists, geneticists, speech therapists, and psychologists were cited as the main difficulties in access, which may indicate an unmet local demand for these professionals in a different way in each country. The other health assessments did not show differences between the two services, indicating similar needs related to the disease and possibly well-managed.

Regardless of the divergences involving the definitions of diagnosis in mental health, the demand for care from professionals such as psychologists, psychiatrists, and neurologists stood out, including difficulty in accessing complete management. The prevalence of diagnosis of psychiatric disorders can be explained by the variable characteristics of the disease, which can include hypotonia, chronic pain, and intellectual deficits, as well as the psychological overload related to the diagnosis of a chronic disease.^1^

Another Brazilian study that evaluated the quality of life of 101 adult individuals with NF1 living in 15 different states identified two main dimensions that impact the appearance of psychic disorders, such as anxiety and depression: uncertainty about the progression of the disease and difficulty in family planning, considering the risk of transmission to offspring.^27^

Although the study was carried out in Brazil, these issues are related to the characteristics of neurofibromatosis type 1 as a chronic multisystem disease that affects all affected individuals regardless of the reality in which they live. Worldwide results indicate rates of around 50 % of anxiety and depression among individuals with NF1.^28^ Thus, the report of difficulty in accessing psychologists and psychiatrists is a concern in both countries, which may be related to a deficit of professionals to meet the demand found in each location, whether for neurologists in Portugal, which has about 655 professionals (about 15,270 inhabitants/professional considering the population of approximately 10 million people),^29^ and psychiatrists in Brazil with approximately 14 thousand professionals (about 15,000 inhabitants/professional considering the population of roughly 200 million people).^30^, ^31^, ^32^

Studies with people with NF1 show that although employment is an indicator of psychological well-being, the uncertainties of the pandemic period led to an increase in stress and anxiety rates among the employed population due to new barriers and job retention challenges, which may justify a greater impact on their clinical management.^33^

Social inclusion in the school and work environment is a stage of great importance in the development and preservation of the autonomy of people with genetic diseases.^25^ Despite reasonable schooling rates and insertion in the labor market, the Brazilian group had a higher rate of reports of learning difficulties, which may be related to the efficiency of the educational system in each country, which has considerably discrepant state investments and different quality indicators. While Portugal, with a population of 10 million inhabitants, invests around 9100 euros per student per year, in Brazil, around 3600 euros are invested annually, directly reflecting on the literacy and school completion rates in each country. Despite this, a Portuguese study observed rates of 48 % of reports of learning difficulties among individuals with NF1.^19^

In addition, in both groups, reports of difficulty in acceptance and bullying in school environments were observed, highlighting how the experience of social insertion can be similar, even in different contexts.^25^ These reports may be related to the very characteristics of the diseases that form cutaneous manifestations, generating discrimination observed in both countries of this study and other studies around the world.^28^ The Brazilian qualitative study indicated prejudice and social marginalization as difficulties with an impact on the quality of life of individuals with NF1, related to the lack of knowledge of the general population and the skin changes resulting from the disease.^27^

This is a self-report study involving individuals with NF1 who have been diagnosed and followed in two different countries. Socioeconomic, cultural, and health literacy acknowledgment are intrinsically different, but the difficulties related to diagnosis and healthcare access in both countries seem to be significant.

The limitations of this study include its small sample size, cross-sectional design, and static presentation of data, which lack a cause-and-effect relationship. Furthermore, data collection was conducted via an electronic form, which created a convenience bias due to the sample being restricted to internet-using individuals. There is a self-report bias, as participants filled in the responses, based on the information they understood about their treatments. Further research is required to draw more robust conclusions. The small sample reflects the disease’s rarity, limiting cross-country comparisons and allowing only an exploratory view of the participating services.

This study is exploratory, and the questions are of a personal, subjective nature and not related to genetic factors. It can be subject to type errors due to the sample size. Moreover, the study focused on a single genetic condition in two metropolitan areas with reference services for clinical genetics, thus not representing the reality of the remaining regions in both countries, but it serves as a starting point to stimulate the development of more concrete approaches to the epidemiological survey and a multidisciplinary approach.

## Conclusions

Despite their differing socioeconomic realities, the exploratory analysis of the sample from a public healthcare service in Brazil and in Portugal revealed similar needs and challenges, including difficulty in accessing early diagnosis, specialized care, longitudinal management of the condition, and social inclusion. Differences in the delivery of medical genetics care between the two countries may reflect divergences in the recognition of access and health literacy. Expanding studies on the prevalence of the disease in both countries would support a more accurate definition of the sample framework and help identify care priorities related to the specificities of NF1 that could improve the quality of life of affected individuals.

## Authors’ contributions

Déborah Domeneghetti de Francisco: Analysis and interpretation of data; Statistical analysis; Writing the article; Critical review of the literature.

Isabela Mayá Wayhs Silva: Conception and design of the study; Data collection, analysis and interpretation of data; Critical review of important intellectual content; Critical review of the literature; Final approval of the final version of the manuscript.

Jorge M. Saraiva: Data collection; Obtaining, analyzing and interpreting data; Effective participation in the supervision of the research; Intellectual participation in propaedeutic and/or therapeutic conduct of cases studied; Critical review of the literature; Final approval of the final version of the manuscript.

Carlos Eduardo Steiner: Critical review of important intellectual content; Intellectual participation in propaedeutic and/or therapeutic conduct of cases studied; Critical review of the literature; Final approval of the final version of the manuscript.

Vera Lúcia Gil da Silva-Lopes: Conception and design of the study; Data collection; Statistical analysis; Critical review of important intellectual content; Obtaining, analyzing and interpreting data; Effective participation in the supervision of the research; Intellectual participation in propaedeutic and/or therapeutic conduct of cases studied; Critical review of the literature; Final approval of the final version of the manuscript.

## Funding

This study was partly funded by the National Council for Scientific and Technological Development (CPNq), Brazil. Process n° 304684/2023-6.

## Data availability statement

The datasets generated and/or analyzed during the current study are available from the corresponding author upon reasonable request.

## Declaration of competing interest

The authors declare no conflicts of interest.
